# The interval between staged bilateral total knee arthroplasties does not affect early complications of the second knee or long-term function of the first and second knees

**DOI:** 10.1186/s12893-024-02442-y

**Published:** 2024-05-14

**Authors:** Feng Ji, Zhenguo Zhao, Lei Zhang, Tongkai Liu, Baoqiang Xu, Wei Li, Shuai Yang, Tianrui Wang

**Affiliations:** 1 Department of Orthopedics, The Affiliated Hospital of Jining Medical University, No. 89, Guhuai Road, Jining, 272000 Shandong China; 2 Institute of Orthopedics, Hebei Medical University Third Hospital, No.139 Ziqiang Road, Shijiazhuang, 050000 Hebei China; 3 Department of Orthopedics, The Affiliated Hospital of Qingdao University, No. 59, Haier Road, Qingdao, 266000 Shandong China

**Keywords:** Staged, Bilateral total knee arthroplasty, Complications, Functional outcome

## Abstract

**Background:**

This study explored the optimal time interval between staged bilateral total knee arthroplasty (BTKA) to minimize early complications of the second TKA and maximise the long-term function of the first and second knees.

**Methods:**

We retrospectively reviewed 266 patients who underwent staged BTKA between 2013 and 2018. Groups 1–4 had time intervals between BTKAs of 1–6, 6–12, 12–18, and 18–24 months, respectively. Demographics, postoperative complications within 90 days of the second TKA, Knee Society Score (KSS), and Western Ontario and McMaster Universities Arthritis Index (WOMAC) score were compared among the groups.

**Results:**

In total, 54, 96, 75, and 41 patients were assigned to groups 1–4, respectively. Although group 1 had the highest overall complication rate (11.11%), there was no significant difference in the complication rate among the four groups. Also, no significant differences were found among the four groups in functional and patient-reported outcomes, in either the first or second knee at 5 years postoperatively, including KSS-knee, KSS-function, WOMAC-pain, WOMAC-stiffness, and WOMAC-physical function. The interval between BTKA did not influence complications or the function of the second knee. The TKA type (posterior-stabilised vs. medial-pivot) and age did not correlate significantly with any scores.

**Conclusions:**

There was no group difference in early complications of the second TKA, and postoperative function was equivalent between the two knees and did not vary by the interval between surgeries. The results of this study give surgeons and patients more choices. If patients cannot tolerate severe symptoms in the contralateral knee after the first TKA, the second TKA should be performed as early as possible. If knee joint function is not well recovered after the first TKA, and patients are anxious to undergo the second TKA, surgeons can advise patients to postpone the operation based on these results.

## Background

Total knee arthroplasty (TKA) is an effective treatment for end-stage knee osteoarthritis (OA), relieving pain and improving joint function and patient quality of life. The number of TKA will increase in the coming decades, with advances in surgical technology and prostheses and the increasing size of the elderly population. One-third of patients with knee OA have bilateral symptoms and up to 19% require bilateral TKA (BTKA) due to severe bilateral knee destruction [1, 2].

BTKA can be performed simultaneously under the same anaesthetic or staged over different hospital admissions. There has been continual debate about the best approach in terms of economics, perioperative complications, and postoperative joint function; no consensus has been reached. Although simultaneous BTKA is superior in terms of length of stay and costs, a growing body of research recommends staged BTKA as a safe, efficacious treatment with fewer complications and less mortality [3–5]. According to the Canadian Hospital Morbidity Database, the annual number of staged BTKA increased by 28%, while simultaneous BTKA decreased by 8% [6].

Currently, the interval between staged BTKA varies and the timing of the second TKA depends largely on patient preference. A literature review found little evidence regarding whether the interval affects the functional outcomes of both knees [7–9]. While three studies have evaluated the optimal timing of the second TKA to minimize early complications, there are no concrete guidelines [9–11].

Therefore, this study explored the optimal interval between staged BTKA to minimize early complications of the second TKA and maximize long-term functional outcomes of both knees.

## Materials and methods

This retrospective cohort study was approved by the Institutional Review Board of the affiliated hospital of Qingdao university. We reviewed the electronic medical record database of arthroplasty patients and identified those who underwent staged BTKA between 2013 and 2018. This database includes clinical and radiological data for patients who underwent annual postoperative follow-ups. The inclusion criteria were a diagnosis of bilateral Kellgren–Lawrence stage III or IV knee OA with related symptoms before the first TKA, staged bilateral primary TKA performed by the same surgeon (not during the same hospitalization), and a minimum postoperative follow-up of 5 years for each knee. Exclusion criteria were posttraumatic or inflammatory arthritis, the need for a constrained prosthesis in either knee, simultaneous BTKA, and staged BTKA during the same hospitalization.

Patients who met the inclusion criteria were divided into groups 1–4 according to the interval between surgeries: 1–6, 6–12, 12–18, and 18–24 months, respectively. The timing of the second TKA was entirely up to the patient and depended on their physical and financial situation (*e*.*g*., ability to tolerate additional pain and limitations in activities of daily living).

The demographic information obtained included gender, age, body mass index (BMI), Charlson Comorbidity Index (CCI), and TKA type. The CCI is calculated by summing the weighted scores for 19 medical conditions to assess underlying comorbidities [12]. The postoperative surgical and medical complications within 90 days of the second TKA used as outcome measures included wound complications, deep vein thrombosis, periprosthetic joint infection (PJI), and urinary, cardiac, pulmonary, and cerebral complications. PJI was identified based on the Musculoskeletal Infection Society definition [13]. Functional and patient-reported outcomes of both knees were assessed preoperatively and 5 years postoperatively using the Knee Society Score (KSS) [14] and Western Ontario and McMaster Universities Arthritis Index (WOMAC) score [15]. The KSS is divided into knee and function scores, both of which range from 0 to 100, with higher scores representing better outcomes. For the WOMAC, a higher score indicates a poorer condition. There are 24 questions and three subscales: pain (5 questions), stiffness (2 questions), and physical function (17 questions).

Although the TKA procedures were performed by five experienced arthroplasty surgeons, all patients received the same prosthesis, which was placed in both knees by the same surgeon. Two different total knee systems were used: posterior-stabilized (PS; NexGen; Zimmer, Warsaw, IN, USA) and medial-pivot (MP; Advance, MicroPort, Arlington, TN, USA). All patients underwent general anaesthesia combined with peripheral nerve blocks, using a thigh tourniquet inflated to 100 mm Hg above the systolic pressure, an anterior midline skin incision, and a medial parapatellar or mid-vastus approach. Patellar arthroplasty was not done in any patient, although peripatellar osteophytes were routinely trimmed following denervation. A drainage tube was placed before skin closure and removed 24–48 h postoperatively.

All patients underwent the same postoperative management, including 48 h of intravenous cefazolin or clindamycin to prevent infection (started 1 h before the skin incision), 10 mg oral rivaroxaban once daily for 30 days to prevent venous thrombus embolism (started within 12–24 h postoperatively), continuous passive motion exercises starting on the first postoperative day, and full weight-bearing and walking with a walking-aid on the second postoperative day.

### Statistical analysis

The statistical analyses were performed using SPSS 23.0 (IBM Corp., Armonk, NY, USA). The normality of the data distribution was determined by the Kolmogorov–Smirnov test. Continuous and categorical variables are expressed as means and standard deviations, and frequencies and percentages, respectively. The paired *t*-test was used to compare continuous variables between the first and second knees. One-way analysis of variance was used to compare continuous variables among groups. The chi-square test was used to compare categorical variables. Correlations were assessed using the Pearson coefficient. *P* < 0.05 indicated statistical significance.

## Results

This retrospective cohort study enrolled 266 patients who underwent staged BTKA, 54, 96, 75, and 41 were done with intervals of 1–6 (group 1), 6–12 (group 2), 12–18 (group 3), and 18–24 (group 4) months, respectively. Table 1 summarises the preoperative data. There was no significant group difference in gender, age, BMI, CCI, TKA type, KSS, or WOMAC score (all *p* > 0.05), except the KSS-knee score for the second knee (*p* = 0.002). In group 4, there were significant differences between the first and second knees for all preoperative scores (all *p* < 0.05), and for KSS-function (*p* = 0.069) and WOMAC-physical function (*p* = 0.179).

**Table 1:** Preoperative data between groups and between the first and second knees

	Group 1 (*n* = 54)	Group 2 (*n* = 96)	Group 3 (*n* = 75)	Group 4 (*n* = 41)	*P* value
Gender (F/M)					
Female (n, %)	47 (87.04)	80 (83.33)	64 (85.33)	33 (80.49)	0.831
Male (n, %)	7 (12.96)	16 (16.67)	11 (14.67)	8 (19.51)	
Age (year)	67.85 ± 8.31	68.90 ± 7.80	70.43 ± 7.25	68.42 ± 7.68	0.266
BMI (kg/m²)					
1st knee	26.74 ± 4.04	26.22 ± 5.00	26.81 ± 4.75	27.33 ± 5.80	0.654
2nd knee	27.03 ± 4.04	26.35 ± 5.02	26.97 ± 4.82	27.15 ± 5.71	0.745
*P* value	0.701	0.858	0.837	0.890	
CCI	2.57 ± 1.24	2.33 ± 1.38	2.52 ± 1.35	2.29 ± 1.40	0.600
The TKA types					
MP (n, %)	32 (59.26)	62 (64.58)	40 (53.33)	24 (58.54)	0.531
PS (n, %)	22 (40.74)	34 (35.42)	35 (46.67)	17 (41.46)	
KSS-knee					
1st knee	36.80 ± 11.22	38.26 ± 10.74	37.87 ± 11.49	38.88 ± 13.08	0.827
2nd knee	38.22 ± 10.55	39.79 ± 9.80	42.91 ± 7.79	44.37 ± 7.30	0.002
*P* value	0.498	0.303	0.002	0.022	
KSS-function					
1st knee	38.33 ± 13.21	40.47 ± 13.67	39.20 ± 13.15	40.12 ± 14.25	0.802
2nd knee	39.54 ± 12.41	41.88 ± 12.74	42.27 ± 10.50	45.24 ± 10.66	0.140
*P* value	0.627	0.462	0.117	0.069	
WOMAC-pain					
1st knee	17.96 ± 4.47	16.80 ± 3.82	17.39 ± 3.82	16.85 ± 4.29	0.345
2nd knee	17.13 ± 4.46	16.41 ± 3.71	16.79 ± 3.51	15.12 ± 3.47	0.063
*P* value	0.334	0.467	0.318	0.048	
WOMAC-stiffness					
1st knee	6.94 ± 1.84	7.28 ± 2.11	7.15 ± 2.06	6.83 ± 2.04	0.605
2nd knee	6.39 ± 1.72	6.63 ± 1.88	6.47 ± 1.72	5.95 ± 1.63	0.239
*P* value	0.108	0.024	0.030	0.034	
WOMAC-physical function					
1st knee	57.48 ± 13.64	55.69 ± 14.25	57.24 ± 13.87	56.49 ± 13.61	0.852
2nd knee	56.11 ± 13.32	54.34 ± 14.18	54.45 ± 12.56	52.66 ± 13.01	0.666
*P* value	0.598	0.513	0.199	0.197	

BMI: body mass index, TKA: total knee arthroplasty, KSS: Knee Society Score, WOMAC: Western Ontario and McMaster Universities Arthritis Index

Table 2 summarises complications within 90 days of the second TKA. Nineteen complications were identified after the second TKA, including six urinary complications (31.58%), four wound complications (21.05%), two PJIs, cardiac complications, pulmonary complications, cerebral complications, and one deep vein thrombosis. Although group 1 had the highest overall complication rate, of 11.11%, there was no significant difference in the rate of complications among the four groups (*p* = 0.582).

**Table 2:** Complications within 90 days of the second total knee arthroplasty between groups

complications (*n*, %)	Group 1 (*n* = 54)	Group 2 (*n* = 96)	Group 3 (*n* = 75)	Group 4 (*n* = 41)	*P* value
Wound complications	1 (1.85)	2 (2.08)	0	1 (2.44)	0.652
DVT	1 (1.85)	0	0	0	0.270
PJI	1 (1.85)	1 (1.04)	0	0	0.605
Urinary	2 (3.70)	2 (3.13)	2 (2.67)	0	0.679
Cardiac	0	1 (1.04)	1 (1.33)	0	0.764
Pulmonary	1 (1.85)	0	0	1 (2.44)	0.294
Cerebral	0	1 (2.08)	1 (1.33)	0	0.764
Overall complications	6 (11.11)	7 (7.29)	4 (5.33)	2 (4.88)	0.582

DVT: deep vein thrombosis, PJI: periprosthetic joint infection

The postoperative scores of four patients (two in group 2 and one each in groups 1 and 4) were not determined because PJI occurred in two patients each within and after more than 90 days following the second TKA. All knees in all groups improved from preoperatively to 5 years postoperatively. Table 3 shows the postoperative outcomes of the four groups, and compares the outcomes between the first and second knees. There were no significant group differences in KSS-pain, KSS-function, WOMAC-pain, WOMAC-stiffness, or WOMAC-physical function in either the first or second knees (all *p* > 0.05).

**Table 3:** Postoperative outcomes between interval groups and between the first and second knee

	Group 1 (*n* = 53)	Group 2 (*n* = 94)	Group 3 (*n* = 75)	Group 4 (*n* = 40)	*P* value
KSS-knee score					
1st knee	86.40 ± 6.53	85.33 ± 6.63	85.76 ± 6.37	86.15 ± 6.14	0.785
2nd knee	85.89 ± 6.50	86.03 ± 6.15	86.72 ± 5.60	87.45 ± 5.53	0.534
*P* value	0.688	0.452	0.329	0.323	
KSS-function score					
1st knee	86.79 ± 9.36	86.17 ± 8.56	85.47 ± 8.74	87.25 ± 8.16	0.719
2nd knee	87.74 ± 8.24	87.77 ± 7.92	86.80 ± 8.57	86.50 ± 8.02	0.773
*P* value	0.583	0.186	0.347	0.680	
WOMAC-pain					
1st knee	4.49 ± 1.40	4.73 ± 1.46	4.65 ± 1.38	4.25 ± 1.72	0.332
2nd knee	4.28 ± 1.47	4.40 ± 1.50	4.47 ± 1.47	3.95 ± 1.54	0.322
*P* value	0.458	0.128	0.425	0.413	
WOMAC-stiffness					
1st knee	1.91 ± 1.08	2.27 ± 1.18	2.11 ± 1.17	2.35 ± 1.23	0.214
2nd knee	2.11 ± 0.99	2.21 ± 1.23	1.96 ± 1.17	2.25 ± 1.06	0.459
*P* value	0.305	0.763	0.443	0.698	
WOMAC-physical function					
1st knee	18.32 ± 4.89	19.65 ± 4.24	19.95 ± 4.56	18.78 ± 4.67	0.169
2nd knee	17.98 ± 4.69	18.69 ± 3.98	19.37 ± 4.15	18.95 ± 3.86	0.312
*P* value	0.716	0.112	0.422	0.855	

KSS: Knee Society Score, WOMAC: Western Ontario and McMaster Universities Arthritis Index

The interval between surgeries did not influence complications (*r* = − 0.080, *p* = 0.192), KSS-knee (*r* = 0.088, *p* = 0.156), KSS-function (*r* = − 0.059, *p* = 0.342), WOMAC-pain (*r* = − 0.035, *p* = 0.574), WOMAC-stiffness (*r* = − 0.012, *p* = 0.845), or WOMAC-physical function (*r* = − 0.092, *p* = 0.139) of the second knee postoperatively. The TKA type (MP or PS) did not correlate with postoperative KSS-knee (*r* = 0.02, *p* = 0.974 and *r* = 0.013, *p* = 0.828, respectively) or KSS-function (*r* = 0.002, *p* = 0.968 and *r* = 0.014, *p* = 0.823, respectively) for either the first or second TKA. Likewise, the age at the time of the first TKA did not affect the KSS-function (*r* = − 0.081, *p* = 0.190 and *r* = 0.040, *p* = 0.518) or WOMAC-physical function (*r* = 0.080, *p* = 0.198 and *r* = 0.041, *p* = 0.509) in either knee.

## Discussion

As a standard, successful surgery, BTKA accounts for a considerable proportion of all TKA performed to alleviate pain and improve physical function in patients with bilateral knee OA. In such patients, BTKA can be performed either simultaneously or as a staged procedure with a variable interval between surgeries. Compared with staged BTKA, simultaneous BTKA is associated with greater blood loss and a higher risk of medical complications and mortality [5]. However, there is no established optimal time frame for performing the second surgery in staged BTKA, and this issue has not garnered much research attention.

One of the concerns regarding a short interval between staged surgeries is the possible increased risk of complications at the time of the second TKA, especially in patients with pre-existing cardiopulmonary disease or advanced age. The first finding of this study was that early complications of the second TKA did not differ significantly among time intervals. The results were similar to previously reported findings. Yeh et al. found a non-significant trend toward higher complication and 90-day readmission rates when the second TKA was performed after 31–90 days [9]. Chen et al. could not identify a safe time frame for performing the second TKA, as the frequencies of complications among various time intervals did not differ significantly [10]. However, those two studies enrolled only patients who had the second TKA within 365 days after the first, even though 36% of patients with bilateral knee OA have contralateral surgery following unilateral TKA after a longer interval [16]. Ishii et al. reported that the median interval between the first and second staged BTKA operations was 12.5 months [17]. Crawford et al. evaluated shorter times between surgeries of 3–6, 7–12, 13–24, and > 24 weeks in 1,005 patients who underwent staged BTKA [11]. They concluded that the time interval did not affect early medical or surgical complications after the second TKA and that it is safe to proceed to the second stage at any time as long as the patients are medically stable. Notably, when comparing simultaneous and staged BTKA, Ritter et al. found that complications did not differ among time intervals, although staged bilateral BTKA at 3–6 months had the lowest mortality rate and fewest disadvantages [18].

The second finding of this study was that the long-term functions of the first and second knees were not significantly different for any time interval. Many studies of staged BTKA have shown that patients have inferior postoperative functional scores for the second-operated knee compared with the first in the short term [19–21]. Poultsides et al. [22]. reported that patients’ preoperative expectations increased for the second surgery compared with the first. This might be one of the main reasons for the poorer clinical outcomes of the second knee. Conversely, Qutob et al. reported that patients with bilateral knee OA commonly elect to have the most symptomatic knee done first [19]. Different symptoms between the two knees lead to different degrees of postoperative improvement, which in turn subjectively influences patient functional scores. In a meta-analysis comparing the clinical outcomes of staged BTKAs, Malahias et al. posited that any postoperative differences between the first and second knees would disappear with longer follow-up. The results of this study, and those of Lizaur-Utrilla et al. [7], confirm that hypothesis. Similar to this study, Lizaur-Utrilla et al. showed that BTKAs performed at different intervals yielded equivalent function scores between the knees at the 5-year follow-up, although the “mental score” and patient satisfaction were better for the second TKA.

Age and TKA type (PS vs. MP) did not influence the outcomes. A study comparing simultaneous and staged BTKA reported that age did not affect the postoperative Oxford Knee Score in either knee [23]. Several studies reported no significant differences in clinical results between the two prosthesis types among patients undergoing unilateral TKA [24, 25]. No study has analyzed whether these two different prostheses influence outcomes in patients with staged BTKA at different intervals. Lizaur-Utrilla et al. found no significant differences in KSS or WOMAC scores between cruciate-retaining and PS TKA [7].

This study had several limitations that should be considered. Firstly, the study was retrospective; the complications of patients admitted to other medical institutions may not have been captured. Secondly, we included a relatively small sample, as the patients were recruited from a single hospital. Therefore, the study may have been underpowered. Hence, it will be necessary to conduct a multicentre, prospective, cohort trial to generate higher-level evidence to confirm our findings. In addition to staged and simultaneous BTKA, BTKA also can be staggered by a few days during a single hospitalization. A recent systematic review reported that the use of staggered BTKA is continuing to decline, as it does not appear to confer a clinical advantage over simultaneous procedures [26]. Despite the lower total cost of the staggered BTKA method compared to staged BTKA, more research needs to explore whether it is safer and confers clinical advantages.

## Conclusion

This study found no differences in early complications of the second TKA according to whether staged BTKA was performed after a short or long interval. The postoperative long-term function of both knees was also equivalent and unaffected by the length of the surgical interval. Furthermore, age and types of prostheses did not influence the outcomes. Our results can help surgeons discuss the timing of the second TKA with patients, which should be based on their preferences. If patients cannot tolerate severe symptoms in the contralateral knee after the first TKA, the second TKA should be performed as early as possible. If knee joint function is not well recovered after the first TKA, and patients are anxious to undergo the second TKA, surgeons can advise patients to postpone the operation based on these results.

## Data Availability

The final dataset will be available from the corresponding author.

## Abbreviations

BTKA: Bilateral total knee arthroplasty
OA: Osteoarthritis
KSS: Knee Society Score
WOMAC: Western Ontario and McMaster Universities Arthritis Index
BMI: Body mass index
CCI: Charlson Comorbidity Index
PJI: Periprosthetic joint infection

## References

[1] Lützner J, Hübel U, Kirschner S, Günther KP, Krummenauer F (2011). [Long-term results in total knee arthroplasty. A meta-analysis of revision rates and functional outcome]. Der Chirurg; Z fur alle Gebiete Der Operativen Medizen.

[2] Memtsoudis SG, Ma Y, Chiu YL, Poultsides L, Gonzalez Della Valle A, Mazumdar M (2011). Bilateral total knee arthroplasty: risk factors for major morbidity and mortality. Anesth Analg.

[3] Fu D, Li G, Chen K, Zeng H, Zhang X, Cai Z (2013). Comparison of clinical outcome between simultaneous-bilateral and staged-bilateral total knee arthroplasty: a systematic review of retrospective studies. J Arthroplast.

[4] Tsay EL, Grace TR, Vail T, Ward D (2019). Bilateral simultaneous vs staged total knee arthroplasty: minimal difference in Perioperative risks. J Arthroplast.

[5] Hu J, Liu Y, Lv Z, Li X, Qin X, Fan W (2011). Mortality and morbidity associated with simultaneous bilateral or staged bilateral total knee arthroplasty: a meta-analysis. Arch Orthop Trauma Surg.

[6] Bohm ER, Molodianovitsh K, Dragan A, Zhu N, Webster G, Masri B, Schemitsch E, Dunbar M (2016). Outcomes of unilateral and bilateral total knee arthroplasty in 238,373 patients. Acta Orthop.

[7] Lizaur-Utrilla A, Serna-Berna R, Vizcaya-Moreno MF, Martinez-Mendez D, Marco-Gomez L, Lopez-Prats FA (2018). Comparison of functional outcomes between the first and second knee in staged bilateral total knee arthroplasty with diverse intervals between stages. J Arthroplast.

[8] Yilmaz B, Sirin E, Kilinc BE, Ozdemir G, Komur B, Heybeli N (2020). Investigation of the Optimal Interval for Staged Total Knee Arthroplasty for the treatment of advanced bilateral gonarthrosis. Acta Chirurgiae Orthopaedicae et traumatologiae Cechoslovaca.

[9] Yeh JZY, Chen JY, Lee WC, Chong HC, Pang HN, Tay DKJ, Chia SL, Lo NN, Yeo SJ (2017). Identifying an Ideal Time Frame for staged bilateral total knee arthroplasty to maximize functional outcome. J Knee Surg.

[10] Chen AF, Rasouli MR, Vegari DN, Huang RC, Maltenfort MG, Parvizi J (2015). Staged bilateral total knee arthroplasty: time of the second side. J Knee Surg.

[11] Crawford DA, Adams JB, Hurst JM, Morris MJ, Berend KR, Lombardi AV (2021). Interval between staged bilateral total knee Arthroplasties does not affect Early Medical or Surgical complications. J Arthroplast.

[12] Charlson ME, Pompei P, Ales KL, MacKenzie CR (1987). A new method of classifying prognostic comorbidity in longitudinal studies: development and validation. J Chronic Dis.

[13] Parvizi J, Gehrke T (2014). Definition of periprosthetic joint infection. J Arthroplast.

[14] Insall JN, Dorr LD, Scott RD, Scott WN. Rationale of the Knee Society clinical rating system. Clin Orthop Relat Res. 1989;248:13–4. 2805470

[15] Whitehouse SL, Lingard EA, Katz JN, Learmonth ID (2003). Development and testing of a reduced WOMAC function scale. J bone Joint Surg Br Volume.

[16] Walmsley P, Murray A, Brenkel IJ (2006). The practice of bilateral, simultaneous total knee replacement in Scotland over the last decade. Data from the Scottish arthroplasty project. Knee.

[17] Ishii Y, Noguchi H, Takeda M, Sato J, Toyabe S (2014). Time between the first and second operations for staged total knee arthroplasties when the interval is determined by the patient. Knee.

[18] Ritter M, Mamlin LA, Melfi CA, Katz BP, Freund DA, Arthur DS. Outcome implications for the timing of bilateral total knee arthroplasties. Clin Orthop Relat Res 1997(345):99–105. 9418626

[19] Qutob M, Winemaker M, Petruccelli D, de Beer J (2013). Staged bilateral total knee arthroplasty: does history dictate the future?. J Arthroplast.

[20] Kim MH, Nahm FS, Kim TK, Chang MJ, Do SH (2014). Comparison of postoperative pain in the first and second knee in staged bilateral total knee arthroplasty: clinical evidence of enhanced pain sensitivity after surgical injury. Pain.

[21] Scott CE, Murray RC, MacDonald DJ, Biant LC. Staged bilateral total knee replacement: changes in expectations and outcomes between the first and second operations. bone Joint J 2014, 96–b(6):752–758. 10.1302/0301-620X.96B6.32793 24891574

[22] Poultsides LA, Ghomrawi HM, Lyman S, Aharonoff GB, Mancuso CA, Sculco TP (2012). Change in preoperative expectations in patients undergoing staged bilateral primary total knee or total hip arthroplasty. J Arthroplast.

[23] Abram SG, Nicol F, Spencer SJ (2016). Patient reported outcomes in three hundred and twenty eight bilateral total knee replacement cases (simultaneous versus staged arthroplasty) using the Oxford knee score. Int Orthop.

[24] Shi W, Jiang Y, Wang C, Zhang H, Wang Y, Li T (2020). Comparative study on mid- and long-term clinical effects of medial pivot prosthesis and posterior-stabilized prosthesis after total knee arthroplasty. J Orthop Surg Res.

[25] Kulshrestha V, Sood M, Kanade S, Kumar S, Datta B, Mittal G (2020). Early outcomes of medial pivot total knee arthroplasty compared to posterior-stabilized design: a Randomized Controlled Trial. Clin Orthop Surg.

[26] Malahias MA, Gu A, De Martino I, Selemon NA, Ast MP, Sculco PK. Staggered bilateral total knee arthroplasty during a single hospitalization: is it still an option? A systematic review. Musculoskelet Surg 2021. 10.1007/s12306-021-00696-w 33721261

